# Structure and Oxidative Folding of AAI, the Major Alfa-Amylase Inhibitor From Amaranth Seeds

**DOI:** 10.3389/fchem.2020.00180

**Published:** 2020-03-17

**Authors:** János Juhász, Zoltán Gáspári, Sándor Pongor

**Affiliations:** ^1^Faculty of Information Technology and Bionics, Pázmány Péter Catholic University, Budapest, Hungary; ^2^3in-PPCU Research Group, Faculty of Information Technology and Bionics, Pázmány Péter Catholic University, Esztergom, Hungary

**Keywords:** AAI, Amaranth alpha-amylase inhibitor, Amaranthus hypocondriacus, oxidative folding, folding intermediate, vicinal disulfide

## Abstract

AAI, the major alpha-amylase inhibitor (AAI) present in the seeds of the Mexican crop plant *Amaranthus hypocondriacus* is a 32-residue-long polypeptide with three disulfide bridges. Its structure is most closely related to the plant amylase inhibitor subfamily of knottins characterized by a topological knot formed by one disulfide bridge threading through a loop formed by the peptide chain as well as a short three-stranded beta sandwich core. AAI is specific against insect amylases and does not act on corresponding human or mammalian enzymes. It was found that the oxidative folding of AAI seems to follow a hirudine-like pathway with many non-native intermediates, but notably it proceeds through a major folding intermediate (MFI) that contains a vicinal disulfide bridge. Based on a review of the pertinent literature, the known vicinal disulfides in native proteins as well as well as the network of disulfide interchanges, we propose that MFI is a kinetic trap corresponding to a compact molten globule-like state which constrains the peptide chain to a smaller number of conformations that in turn can be rapidly funneled toward the native state.

## AAI: The Major Alpha Amylase Inhibitor of *Amaranthus hypochondriacus*

AAI (amaranth amylase inhibitor) is an alpha-amylase inhibitor isolated from the Mexican crop plant *Amaranthus hypochondriacus*. Amaranth grains are known to be cultivated for about 8,000 years and were part of the diet of the Aztecs. Today, Amaranth is grown in Mexico, Peru, and Bolivia, but the world's largest producer is China with an estimated annual production of 87 million metric tons. Amaranths are classified as pseudo cereals as they are not in the same botanical family as true cereals to which their grains bear similarity.

**Figure 1:**
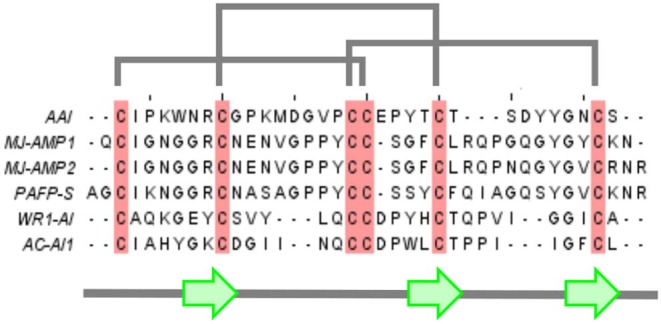
Multiple alignment of AAI with other members of the amylase inhibitor knottin family. Mj-AMP1, Mj-AMP2: *Mirabilis jalapa L*. seed antimicrobial peptides 1 and 2 (Cammue et al., 1992), PAFP-S: *Phytolacca americana* seed antifungal peptide (Gao et al., 2001), WR-AI1: *Wrightia religiosa* cystine knot α-amylase inhibitor (Nguyen et al., 2014), AC-AI1: *Allamanda cathartica* cystine knot α-amylase inhibitor (Nguyen et al., 2015).

Aqueous extracts of Amaranth grains were found to inhibit insect alpha-amylases extracted from the larvae of the red flour beetle (*Dibolium castaneum*) and of the grain borer (*Prostephanus truncatus*). The alpha-amylase was purified with classical techniques (Chagolla-Lopez et al., 1994). Briefly, crude extracts of amaranth flour were fractionated by ammonium sulfate precipitation (35–65%), fractionated on G75 Sepharose columns and the lyophilized active fractions were subjected to ion exchange chromatography The majority of inhibitory activity was found in one major peak which was further purified with reverse phase HPLC (RP-HPLC). The inhibitory activity was resistant to heat. As amino acid analysis revealed a high percentage of cysteine with no free sulfhydryl groups, the samples were reduced and pyridylethylated prior to sequencing. Digestion with trypsin and cyanogen bromide resulted in 7 overlapping peptides sequenced by automated Edman degradation which gave an assembled sequence of 32 amino acids with 6 cysteines and four prolines. The disulfide bridges were determined from partial double digests of the non-reduced peptide obtained with trypsin/chymotripsin. The disulfide topology could be determined except for the uncertainty caused by the vicinal cysteines 18 and 19, but a consensus topology could be predicted based on the multiple alignment (Figure 1) which was subsequently confirmed with both NMR (Lu et al., 1999) and X-ray crystallography (Pereira et al., 1999).

**Figure 2:**
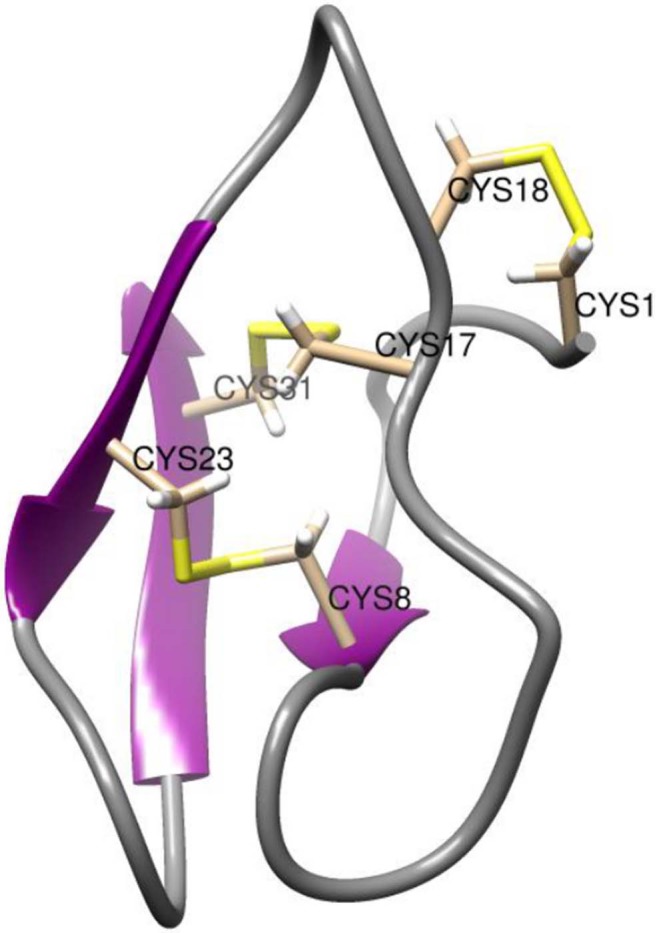
NMR structure of AAI. The NMR structure is deposited in the PDB under the id 1QFD (Lu et al., 1999). All six cysteine residues are labeled. The vicinal cysteines Cys17-Cys18 are on the top right in this view. Structural representations were prepared with Chimera (original figure based on publicly available data) (Pettersen et al., 2004).

As shown by the NMR structure in Figure 2, AAI contains 3 disulfide bridges in the *abcabc* topology, bridge *a* connecting Cys1 and Cys18, bridge *b* connecting Cys8 and Cys23, and bridge c connecting Cys17 and Cys31, respectively. With its length of only 32 residues, AAI was the shortest alpha-amylase inhibitor known at the time of its discovery, about 10 years later related amylase inhibitors of 30 amino acids were discovered (Tam et al., 2015). Purified AAI was found to be specific for insect amylases but not inhibiting mammalian amylases. This is an important property since many edible high protein seeds such as those of legumes contain enzyme inhibitors that are toxic to animals and humans and have to be destroyed by cooking or roasting.

## The 3D Structure of AAI

The 3D structure of AAI was first predicted with molecular modeling based on homology to other peptides (Chagolla-Lopez et al., 1994). Namely, multiple alignment (Figure 1) revealed that AAI is homologous to knottin proteins noted for a pseudo-knot formed by a conserved disulfide arrangement found in a family of short peptides. The term was coined—as far as we know—a few years before the isolation of AAI (Le Nguyen et al., 1990). As of today there is well-maintained database of knottin structures (knottin dbase) that currently has 3,320 sequences and 214 3D structure entries (Postic et al., 2018) (www.dsimb.inserm.fr/KNOTTIN/). Based on the abundant structural information available today, we can safely conclude that AAI belongs to a specific subclass of knottins, the plant alfa- amylase inhibitors (Tam et al., 2015) that contain a short beta sandwich of three (sometimes only two) beta strands, the third strand being sometimes less regular due to the shortness of the sandwich. The short beta sandwich was included into the first predicted structure of AAI which in this way turned out to be analogous to the consensus structure of knottins, more exactly to the subgroup of plant alpha-amylase inhibitor knottins defined later (Tam et al., 2015).

**Figure 3:**
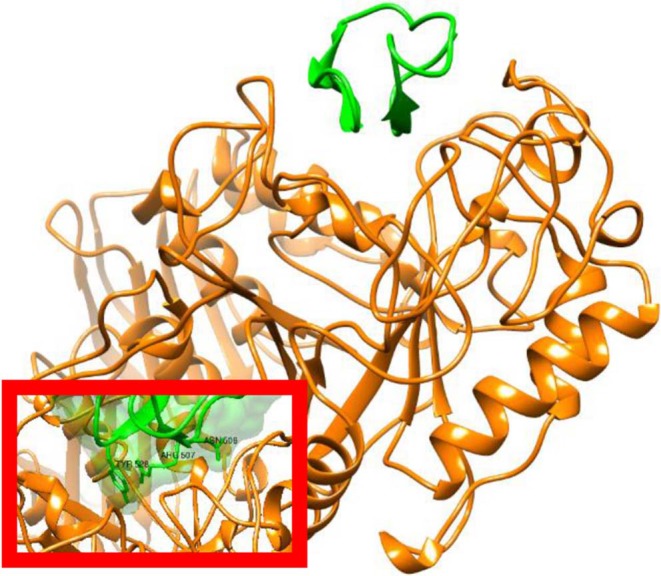
X-ray structure of AAI in complex with the *Tenebrio molitor* alpha-amylase enzyme. Figure prepared from PDB entry 1CLV (Pereira et al., 1999). The amylase is shown in orange and the inhibitor in green with ribbon representation. The inset shows a surface representation of AAI highlighting the insertion of the inhibitor in to the substrate binding cleft of the enzyme. Structural representations were prepared with Chimera (original figures based on published data) (Pettersen et al., 2004).

The X-ray structure of AAI, in complex with the α-amylase of yellow meal worm (*Tenebrio molitor*) larvae (TMA) was determined at 2.0 Å resolution (Pereira et al., 1999). In addition to confirming the knottin-like structure, it was found that AAI binds to the active-groove of TMA via two segments, the first and the last intercysteine loops. According to the X-ray structure, TMA-bound AAI blocks the central four sugar-binding subsites of TMA rendering substrate binding impossible (Figure 3). In addition, molecular docking techniques were used predict the binding of AAI to porcine pancreatic alpha-amylase (PPA). It was found AAI can form only 8 direct (not solvent-mediated) hydrogen bonds to the porcine enzyme as opposed to the 14 such TMA, which provides a simple molecular explanation to the inhibitory specificity of AAI toward insect amylases.

## Synthesis and *in vitro* Oxidative Folding of AAI

The AAI peptide was synthesized manually on a 1-mmol scale by solid-phase peptide synthesis using an Fmoc (N-(9-fluorenyl)methoxycarbonyl) methodology (Lozanov et al., 1997; Cemazar et al., 2003). The crude peptide was isolated with yield 90% and purified to homogeneity (98%, RP-HPLC). Disulfide bridges were formed by oxidative folding in a cysteine (1 mM)-cystine (0.05 mM) redox buffer containing 1 M guanidine hydrochloride. The reaction was left to proceed at room temperature for 16^h^, the overall yield of the HPLC purified peptide was >90%. The synthetic product had the same physicochemical and enzyme inhibitory properties as the natural product. In addition, several orthogonal cysteine protection schemes were tried, in which the disulfide bridges were produced in a well-defined order but active inhibitors were not obtained. It was concluded that “…oxidative folding of AAI may not be a simple process but may rather proceed via transient intermediates, i.e., through closing and opening of disulfide bonds” (Lozanov et al., 1997).

**Figure 4:**
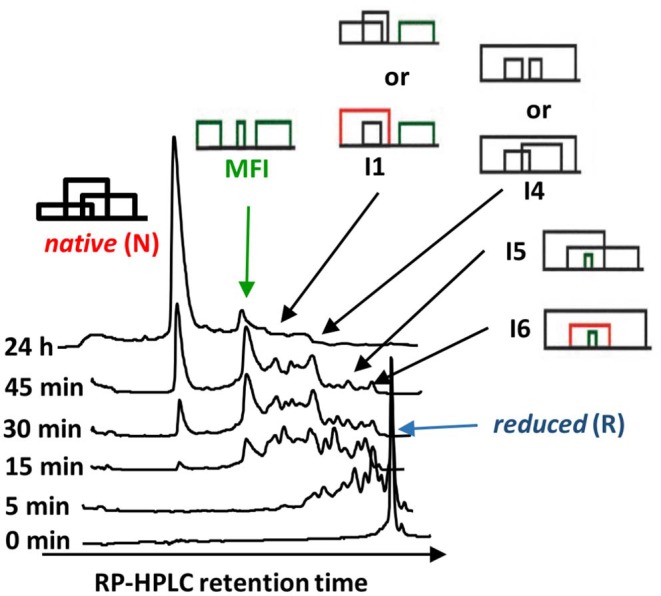
The oxidative folding intermediates of AAI. Oxidative folding was carried out at pH 8.5, 25°C, by placing 100 mg_liter^−1^ AAI into the refolding buffer (100 mM ammonium acetate/1 M guanidinium hydrochloride/1 mM cysteine/0.05 mM cystine/2 mM EDTA). The same distribution of intermediates was observed when the folding was carried out at different pH values (7.0, 7.5, 8.0, and 8.5) and in the absence of guanidinium hydrochloride. The disulfide connectivity of the intermediates was determined by acid trapping and mass spectrometry as described in the text. The disulfide pairings of intermediates I1 and I4 could not be unequivocally determined due to the vicinal cysteines.

**Figure 5:**
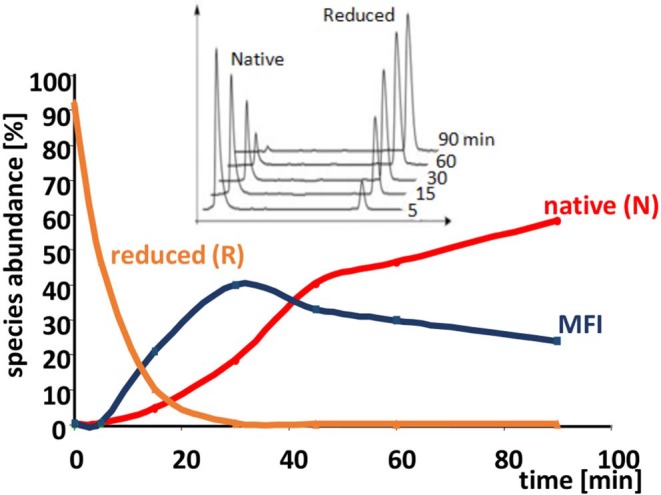
Time course of the oxidative folding of AAI. The abundance of reduced and native forms, as well as the MFI are shown as a function of time during folding. The accumulation of the MFI and then its gradual conversion to the native form is clearly visible. Inset: reductive unfolding of AAI carried out with dithio-treitol (DTT) shows an all-or-none mechanism i.e., no intermediates are shown in addition to the native and the completely reduced species. The reduction was carried out in a buffer similar to that described in the legend to Figure 4 except that it contained 1–10 mM of DTT instead of cysteine/cystine (original figure based on published data).

The oxidative folding of AAI was studied with a variety of techniques [acid quenching followed by RP-HPLC and mass spectrometry, NMR, photoCIDNP [photochemically induced dynamic nuclear polarization], CD [circular dichroism]] (Cemazar et al., 2003, 2004). The conditions included the synthesis conditions mentioned above as well as an enzyme catalyzed folding carried out in the presence of bacterial disulfide isomerase DsBC In most of the cases, a fixed group of 5 fully oxidized intermediates were identified (Figure 4). All but two of the observed disulfide bridges were non-native, and a non-native vicinal disulfide bond between cysteines 17 and 18 was present in three of the intermediates (Figure 4). Because of the vicinal cysteines, the disulfide connectivity could not be unequivocally determined in the case of I1 and I4, so we assigned alternative structures to both of them (I1-1, I1-2 and I4-1, I4-2, respectively). The time course of oxidative folding shows a further peculiarity, i.e., a dominant, major folding intermediate (MFI) that also contained the vicinal disulfide bridge was transiently present (Figure 5). The position of MFI within the chromatogram shows that MFI is relatively hydrophilic as compared to the reduced species which suggests that the hydrophobic residues may be more buried than in the reduced species. Even though native AAI and MFI possess different disulfide pairings, their sizes are indistinguishable within the experimental error (Cemazar et al., 2004). This makes us believe that MFI may play a role similar to that of the molten globule state of larger proteins by constraining the peptide chain to a smaller number of conformations that can be rapidly funneled toward the native state.

**Figure 6:**
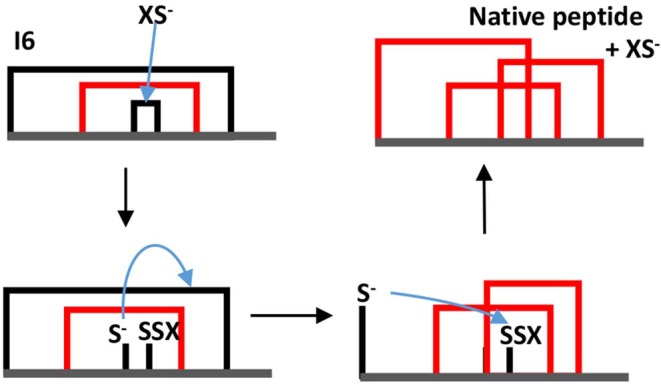
Example of a thiolate-catalyzed intramolecular rearrangement between two disulfide bridges. In this example intermediate I6 is transformed into the native structure. XS^−^ the thiolate form of the redox assistant Cys molecule the blue arrow indicates a nucleophilic attack. Note that in this example the thiolate attacks bridge 3–4 first, but the native structure can be reached also if the thiolate first attacks bridge 1–6.

**Figure 7:**
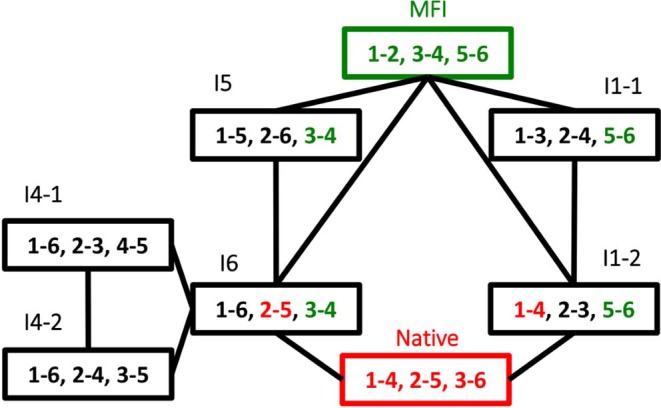
Oxidative folding map built from the disulfide intermediates observed oxidative folding of AAI. Each node is an intermediate, folding map of the observed disulfide intermediates shown in Figure 4. The nodes are the observed intermediates, numbers indicate the serial order of cysteine residues within the sequence, i.e., 1 = Cys1, 2 = Cys8, 3 = Cys17, 6 = Cys30. The edges are intramolecular folding reactions (reshuffling reactions), an example for them is shown in Figure 6.

The dominance of MFI was observed both for the enzyme catalyzed and for the cysteine/cystine catalyzed reaction, the only difference was that in the enzyme catalyzed reaction intermediate I1 was the most intensive for the first minutes of the folding process (data not shown). The disulfide connectivity of MFI is “bead-like” i.e., Cys residues pair with their sequential neighbors. This is in good accordance with the general view that local interactions dominate the first stages of protein folding. In order to get insights into the role the intermediates we prepared a folding map with intermediates as the nodes and disulfide exchange reactions as the edges (Agoston et al., 2005). The logics underlying this representation is that fully oxidized intermediates such as observed for AAI can undergo intramolecular disulfide interchange reactions that rewire the two participating disulfide bridges (example shown in Figure 6). The full network of the intermediates has 15 nodes representing fully oxidized intermediates and 45 edges each representing a rearrangement analogous to that showed in Figure 6. If we map the observed intermediates on this network, we get a folding map shown in Figure 7. One can notice that (i) the network of observed intermediates is small i.e., there is a short route between the intermediates, (ii) MFI plays a central role in this small network as it is connected to 4 out of 6 observed intermediates; and finally, (iii) the native state is accessible via two intermediates, and is only two reshuffling steps away from MFI.

NMR and photoCIDNP experiments revealed that the reduced form R is close to random coil, but MFI has some structure (or rather a set of slowly interconverting structures) that differs from that of the native AAI especially in terms of the accessibility of aromatic side chains that is revealed by photoCIDNP. Time resolved NMR revealed a monotonous change in the aliphatic and aromatic NMR signals, respectively, and confirmed that enzyme catalyzed reaction was somewhat faster, even though it proceeded through the same intermediates, with minor quantitative differences at the first stages of the reaction (Carugo et al., 2003).

## The Role of the Vicinal Disulfide Bridge

**Figure 8:**
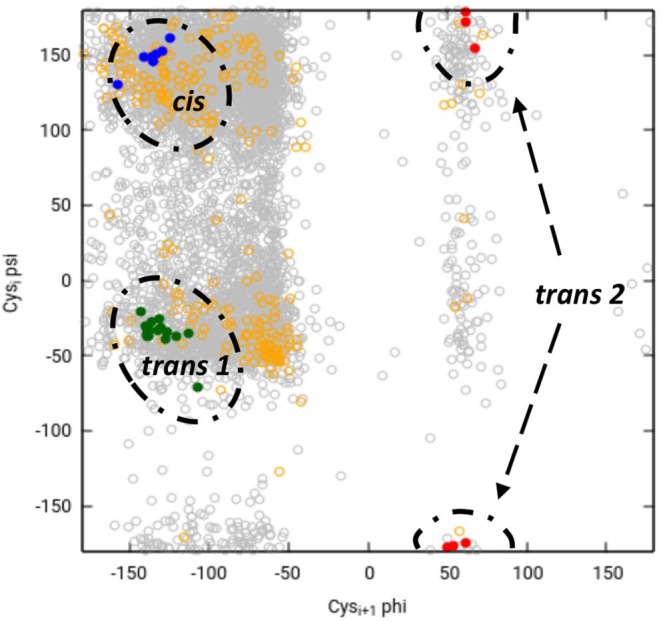
Ramachandran-like representation of vicinal disulfide bridges in current 3DB databases. Data from the Cys.sqlite database (Fobe et al., 2019) filtered according to the CATH 40% non-redundant list (Dawson et al., 2017). Gray circles represent all disulfide-forming cysteines, orange circles depict all neighboring cysteines that are not linked to each other. Vicinal disulfide bridges with *cis* amide bonds between the cysteines are shown with blue dots, whereas the two clusters with *trans* amide bonds are colored green (cluster 1) and red (cluster 2). Note that due to the “−180°–180°” convention of the Ramachandran map, cluster 2 is split and appears at the top and the bottom.

**Figure 9:**
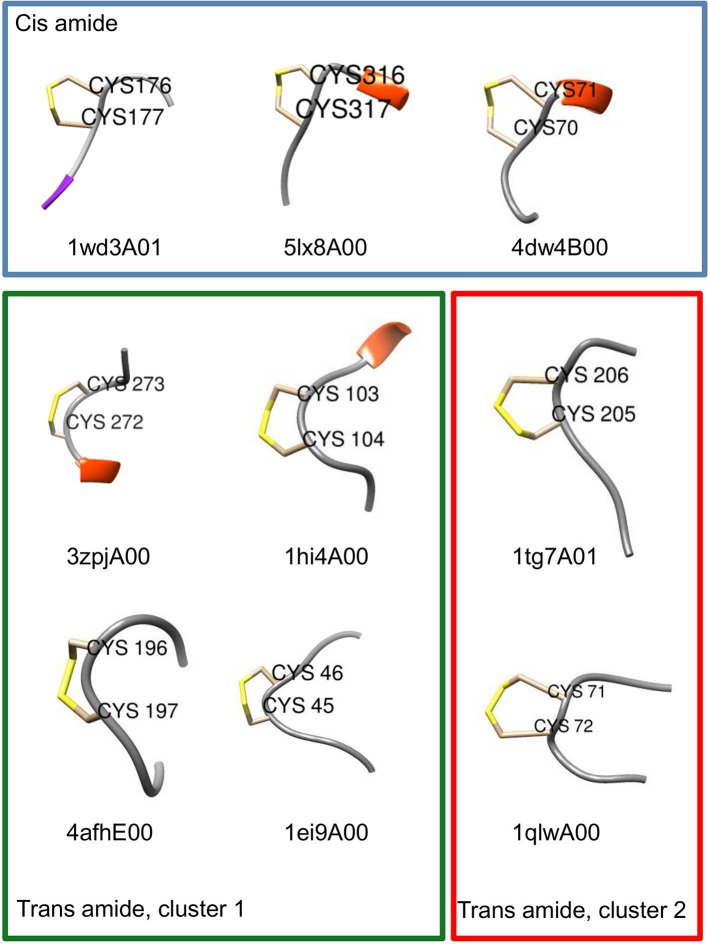
Examples of turn-like conformations induced by vicinal disulfide bridges. Examples for the *cis* and *trans* clusters are shown with CATH domain codes. Six residue fragments re shown with positions 3 and 4 corresponding to the disulfide-forming vicinal cysteines. Structural representations were prepared with Chimera (Pettersen et al., 2004). Frame coloring corresponds to the cluster colors in Figure 8.

Chemical intuition suggests that a vicinal S-S bond (and the associated eight-member ring) could have structural effects similar to a proline residue (a five-member ring) in constraining the movement of the main chain in such a way that a turn is formed (Carugo et al., 2003). Or, as Jane Richardson and associates put it: “A vicinal disulfide acts conformationally rather like a super-sized proline ring that can rigidly organize a connected region of side chains and backbone” (Richardson et al., 2017). About the molecular details of the 8 membered rings there is a variety of opinions. Earlier studies pointed out that the peptide bond of the vicinal disulfide bridge is *trans* and it imposes a turn like structure (Carugo et al., 2003). A detailed theoretical study suggested that *cis*-amide containing rings are also stable, although no examples were found in the databases at the time (Hudáky et al., 2004). A more complete statistical overview of current databases suggests that vicinal disulfide turns form 3 distinct clusters, two corresponding to *trans* amide bond, one to cis (Figure 8). The *cis* vicinal structures depicted on Figure 9 are in fact closest to type VIa turns, but, in agreement with the theoretical calculations (Hudáky et al., 2004), the Cα distance criterion of <7 Å is not met. On the other hand, the majority of the vicinal disulfides belong to the other clusters that resemble a type VIII turn with a distorted *trans* peptide bond between the two cysteines, with omega torsion angles ranging from −176° to −152° with an average of around −162° in our demonstrative data set. Taken together, the pronounced difference observed between the oxidized and reduced states, suggests that vicinal disulfides may act “as a ‘redox-activated' conformational switch” (Carugo et al., 2003).

## Summary and Conclusions

Oxidative folding can be best pictured as the fusion of two competing processes, the formation of covalent disulfide bridges on the one hand, and the formation the non-covalent interactions on the other hand, that are known to give rise to secondary and tertiary structure. It is believed that there are two extreme pathway types of oxidative folding (Narayan et al., 2000; Chang, 2011). The one nicknamed BPTI-like after bovine pancreatic trypsin inhibitor, is dominated by conformational folding, and as a consequence, only (or mostly) native disulfide bridges are found among the observable intermediates. In contrast, “hirudin-type” folding is dominated by disulfide formation and is characterized by a variety of intermediates with mostly non-native disulfide bridges. AAI clearly falls into this latter group, based on the predominance of non-native disulfide bonds in its folding intermediates as well as on the all-or-none type reductive unfolding profile characteristic of this group (Figure 5, inset). As the intermediates seem to be closely related (Figure 7) we tend to believe that AAI can form via a variety of similar pathways, i.e., there may be no single folding pathway that could be clearly distinguished as compared to the others. The dominance of MFI in the process can be explained by analogies to the familiar concepts of protein folding: (i) The beadlike disulfide topology corresponds to local connections that form in the early phases of the conformational folding process. To this we add, that an similar bead-like structure is the most abundant intermediate during the oxidative folding of hirudin core domain (Chang, 2011), (ii) The compact, hydrophilic form (apparent from NMR and RP-HPLC, respectively) as well as the slowly interchanging conformations (NMR) are reminiscent of the molten globule-like state observed in protein folding, (iii) The centrally located vicinal disulfide turn may help the molecule to get into a compact form. Intermediate I6 contains two further disulfide bonds that fix the chain in a roughly U-shaped form. Interestingly, this disulfide arrangement is analogous to the ladder-like intermediate seen in the folding of various other knottins (for reviews see Cemazar et al., 2008; Reinwarth et al., 2013). Summarizing it appears that MFI forms via aspecific collapse and is consolidated into the native state via intramolecular disulfide reshuffling reactions.

To our knowledge, the biotechnological potential of AAI-like proteins is yet a largely unexplored area. Although there are a number of disupfide-rich peptides that are extensively used in protein design as scaffolds (Wang and Craik, 2018) AAI is yet to be explored in this respect. Although defensin-like peptides, containing vicinal cysteines, are among the successful examples where designed disulfide pairings can be synthetically achieved (Zheng et al., 2017) the possible utilization of a folding route with a transient vicinal disulfide has not been investigated. We propose that future protein design attempts could make use of the large interaction surface of AAI as well as its unique folding mechanism to explore novel possible applications and synthetic routes.

## Author Contributions

JJ, ZG, and SP designed the review and drafted the manuscript.

### Conflict of Interest

The authors declare that the research was conducted in the absence of any commercial or financial relationships that could be construed as a potential conflict of interest.
